# Phylogenomics of an extra-Antarctic notothenioid radiation reveals a previously unrecognized lineage and diffuse species boundaries

**DOI:** 10.1186/s12862-019-1345-z

**Published:** 2019-01-10

**Authors:** Santiago G. Ceballos, Marius Roesti, Michael Matschiner, Daniel A. Fernández, Malte Damerau, Reinhold Hanel, Walter Salzburger

**Affiliations:** 1 Instituto de Ciencias Polares, Ambiente y Recursos Naturales (ICPA), Universidad Nacional de Tierra del Fuego (UNTDF), Fuegia Basket 251, V9410BXE Ushuaia, Argentina; 2 Centro Austral de Investigaciones Científicas (CADIC-CONICET), Bernardo A Houssay 200, V9410BXE Ushuaia, Ushuaia, Tierra del Fuego Argentina; 3 Zoological Institute, University of Basel, Basel, Switzerland; 4 Department of Zoology, University of British Columbia, Vancouver, BC Canada; 5 Department of Biosciences, Centre for Ecological and Evolutionary Synthesis (CEES), University of Oslo, Oslo, Norway; 6 Forestry and Fisheries, Institute of Fisheries Ecology, Johann Heinrich von Thünen Institute, Federal Research Institute for Rural Areas, Hamburg, Germany

**Keywords:** RAD, Adaptive radiation, Patagonotothen, Cryptic species

## Abstract

**Background:**

The impressive adaptive radiation of notothenioid fishes in Antarctic waters is generally thought to have been facilitated by an evolutionary key innovation, antifreeze glycoproteins, permitting the rapid evolution of more than 120 species subsequent to the Antarctic glaciation. By way of contrast, the second-most species-rich notothenioid genus, *Patagonotothen*, which is nested within the Antarctic clade of Notothenioidei, is almost exclusively found in the non-Antarctic waters of Patagonia. While the drivers of the diversification of *Patagonotothen* are currently unknown, they are unlikely to be related to antifreeze glycoproteins, given that water temperatures in Patagonia are well above freezing point. Here we performed a phylogenetic analysis based on genome-wide single nucleotide polymorphisms (SNPs) derived from restriction site-associated DNA sequencing (RADseq) in a total of twelve *Patagonotothen* species.

**Results:**

We present a well-supported, time-calibrated phylogenetic hypothesis including closely and distantly related outgroups, confirming the monophyly of the genus *Patagonotothen* with an origin approximately 3 million years ago and the paraphyly of both the sister genus *Lepidonotothen* and the family Notothenidae. Our phylogenomic and population genetic analyses highlight a previously unrecognized linage and provide evidence for shared genetic variation between some closely related species. We also provide a mitochondrial phylogeny showing mitonuclear discordance.

**Conclusions:**

Based on a combination of phylogenomic and population genomic approaches, we provide evidence for the existence of a new, potentially cryptic, *Patagonotothen* species, and demonstrate that genetic boundaries between some closely related species are diffuse, likely due to recent introgression and/or incomplete linage sorting. The detected mitonuclear discordance highlights the limitations of relying on a single locus for species barcoding. In addition, our time-calibrated phylogenetic hypothesis shows that the early burst of diversification roughly coincides with the onset of the intensification of Quaternary glacial cycles and that the rate of species accumulation may have been stepwise rather than constant. Our phylogenetic framework not only advances our understanding of the origin of a high-latitude marine radiation, but also provides the basis for the study of the ecology and life history of the genus *Patagonotothen*, as well as for their conservation and commercial management.

**Electronic supplementary material:**

The online version of this article (10.1186/s12862-019-1345-z) contains supplementary material, which is available to authorized users.

## Background

Species radiations, that are the rapid evolution of taxonomic, ecological and morphological disparity within a clade, have long been recognized as essential models to study organismal diversification [1]. The most extensive case of a fish radiation in the marine environment is represented by the Antarctic clade of the teleost suborder Notothenioidei that has diversified into over 120 species [2–4]. Notothenioids dominate the fish fauna on the Antarctic continental shelves, where they comprise approximately 77% of the fish species diversity and more than 90% of fish biomass [2]. It is assumed that the evolutionary innovation of antifreeze glycoproteins (AFGPs) was key for the successful colonization of cold water niches, which in turn triggered the adaptive radiation of the Antarctic notothenioids [4–8]. It has further been proposed that the Antarctic notothenioid radiation was shaped by paleoclimatic changes that resulted in continuous ecological divergence into recurrently opening niches [6, 9]. Today, the bulk of notothenioid species inhabit the area south of the Antarctic Polar Front.

The notothenioid genus *Patagonotothen*, however, is found almost exclusively in non-Antarctic waters. It is nested within the Antarctic notothenioid clade and represents the second-most species-rich genus of Notothenioidei with 15 recognized species to date (Eastman and Eakin available at https://people.ohio.edu/eastman/; version Dec. 15, 2016), only surpassed in taxonomic diversity by the Antarctic genus *Pogonophryne* with 24 species. All *Patagonotothen* species occur in marine waters around the southern part of South America (Patagonia), the only exception being *P. guntheri*, which has a trans-Antarctic Polar Front range extending from the southern Patagonian Shelf to the Shag Rocks Shelf [10, 11]. Morphological analyses indicate that *P. guntheri* is a derived species within the genus, suggesting that it dispersed southward secondarily [10, 12] and that, therefore, the initial *Patagonotothen* radiation most likely occurred in non-Antarctic waters. The age of the most recent common ancestor of *Patagonotothen* has been estimated to be around 5 Ma [6], making it a relatively recent radiation. The exact drivers of this radiation remain unknown, but are unlikely to be related to the putative evolutionary key innovation of notothenioids, the AFGPs, since the temperature of Patagonian waters is usually well above freezing point [13]. Furthermore, it has been shown that at least some *Patagonotothen* species have secondarily lost the ability to produce AFGPs [4, 7, 14]. Interestingly, an unrelated but similarly species-rich radiation has occurred in the same geographic region in the mollusc genus *Nacella*. In this case, it has been proposed that the currently overlapping distribution of several closely related *Nacella* species constitutes a secondary contact scenario after allopatric speciation, or incipient separation in different refugia during glaciation cycles followed by geographical re-expansion and ecological separation [15]. Whether the same processes also underlie the *Patagonotothen* radiation remains unknown.

In addition to extrinsic environmental factors, intrinsic factors (such as dispersal ability, genetic architecture or chromosomal instability; see [3]) specific to *Patagonotothen* might have played a role in the diversification of this clade. This is suggested, for example, by their comparison with the evolutionary history of *Eleginops maclovinus*, another notothenioid species occurring along the Patagonian coast. *E. maclovinus* is the only representative of the family Eleginopsidae and has an estimated divergence time from the Antarctic clade of around 40 Ma [5]. This species shows strong genetic footprints of past glacial cycles [16, 17]; however, only weak population structure is found along the Pacific and Atlantic Patagonian coast [16, 17]. Thus, while *E. maclovinus* has remained a single taxonomic unit for an extended period of time, *Patagonotothen* have diversified into more than a dozen of species in a relatively short period of time in the same geographic region.

The *Patagonotothen* species are of great abundance throughout the Patagonian shelf [18] and play an important role in the trophic ecology of this region: They consume a variety of benthic and supra-benthic invertebrates [10, 19, 20] and are prey to almost all medium to large-sized fish predators including hakes (*Merluccius* spp.), toothfish (*Dissostichus eleginoides*), kingclip (*Genypterus blacodes*), redcod (*Salilota australis*) and rajids [21]. Some *Patagonotothen* species are or have been subject to extensive exploitation (especially *P. ramsayi* and *P. guntheri*) and represent a significant proportion of the by-catch of bottom trawl fisheries [22, 23]. Despite the ecological and economic importance of *Patagonotothen*, the genus has been little explored with respect to its taxonomy and evolutionary history. One problem is that some of the species are very similar morphologically, making species identification difficult [24–26]. Knowing the taxonomic status, and the number of independent evolutionary units in that genus, is important not only for understanding the *Patagonotothen* radiation, but also to study their ecology and life history, as well as for conservation and management purposes. In addition, the evolutionary history of *Patagonotothen* – that is, the colonization of warmer and thermally more unstable waters from Antarctic ancestors and the subsequent diversification – resembles, at least to some extent, a possible scenario that could apply to other notothenioids in the light of global warming and the associated rise in water temperature in the Southern Ocean.

Most available molecular phylogenetic hypotheses that aimed at resolving the evolutionary relationships among notothenioids and that include *Patagonotothen* species are based on a small number of representatives of this genus and on mitochondrial and/or few nuclear markers, generally yielding low to moderate support for internal phylogenetic nodes [6, 27]. In this study, we report a new time-calibrated phylogenetic hypothesis for *Patagonotothen* that is based on genome-wide single nucleotide polymorphisms (SNPs) identified through restriction site-associated DNA sequencing (RADseq) in twelve *Patagonotothen* species, including a putative new species. We also assessed the level of differentiation between closely related species within the genus *Patagonotothen,* applying a population genomic approach.

## Results

### De novo assembly of RAD loci

We conducted a de novo assembly of RAD loci with the software Stacks, version 1.41 [28] and assessed the performance of parameters following the protocol suggested in reference [29] (see Methods for more details). Briefly, we varied parameters M (distance allowed between stacks) and n (distance allowed between catalogue loci) from 1 to 6 (fixing M = n), and plotted the number of loci shared by at least 80% of a subset of ten samples (Additional file 1). Considering that, for our data set, the number of widely shared loci plateaus starts at about M = 3, we retained this value for the main analysis. In addition, preliminary analyses using only a clade including two replicated samples showed a clustering according to species identity and replicated samples clustered together tightly in a Neighbour-Joining (NJ) tree (not shown) demonstrating that the de novo assembly (with parameters M = *n* = 3) performed with the Stacks pipeline generated robust results.

In the whole set of samples, we obtained 3.9 ± 1.5 million quality-filtered Illumina reads per individual. With these raw data the de novo assembly generated an average of 55,871 ± 8520 loci per individual with a stack depth of 59 ± 20.

### Species assignment and early diversification

**Fig. 1:**
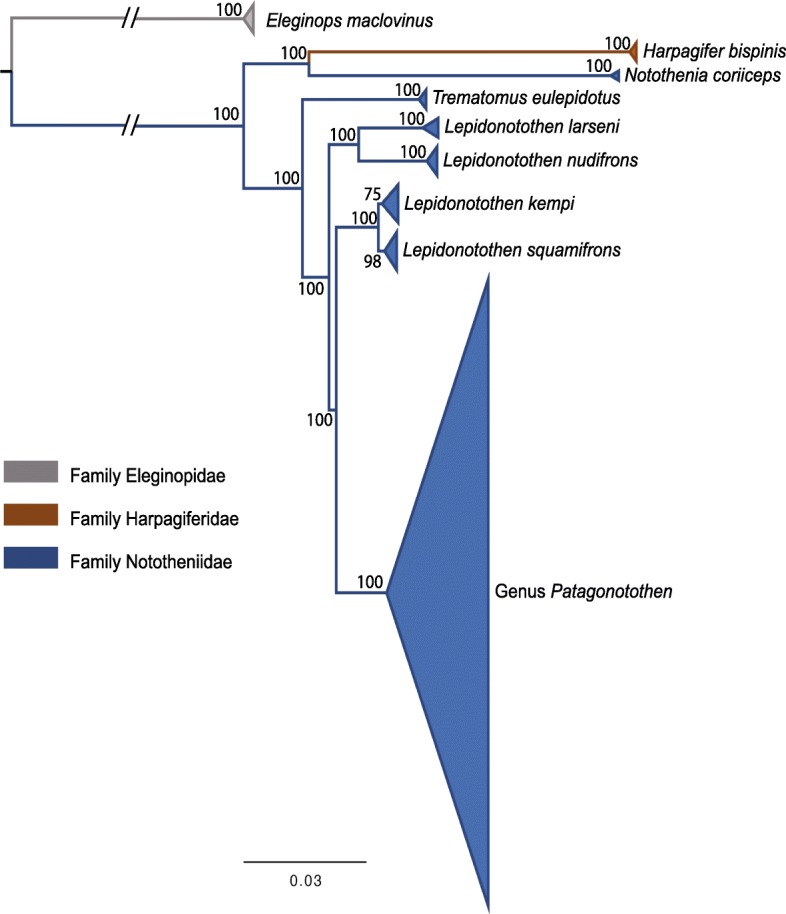
Maximum-Likelihood tree based on 11,804 SNPs from 1682 RAD loci including distant outgroups to assess early diversification in nototheniods. Individuals from the same species in outgroups and from all *Patagonotothen* species have been collapsed for clarity (see full tree in Additional file 1: Figure S1). Node labels represent bootstrap support values as obtained with RAxML

To test for phylogenetic structuring according to morphological species identification, and to evaluate the use of RADseq to resolve early cladogenetic events in this radiation, we first performed a Maximum-Likelihood (ML) phylogenetic analysis using all samples and including all outgroups (Table 1). Allowing for no more than 5% missing data, this analysis was based on 11,804 SNPs across 1682 RAD loci. All samples from the same species formed monophyletic groups as expected, except for three species of the genus *Patagonotothen*: *P. cornucola*, *P. guntheri* and *P. ramsayi* (Additional file 2 and Fig. 1; see following sections for further details). The reconstructed phylogeny supports the paraphyly of the family Nototheniidae as the position of *N. coriiceps* is closer to *H. bispinis* (family Harpagiferidae) than to other members of the Nototheniidae (Fig. 1). The paraphyly of the genus *Lepidonotothen* is also supported, with *L. kempi* and *L. squamifrons* being more closely related to the genus *Patagonotothen* (Fig. 1) than *L. larseni* and *L. nudifrons*. Our finding of paraphyly of both Nototheniidae and *Lepidonotothen* is in agreement with previous phylogenetic studies of notothenioids based on smaller data sets [5, 6, 27, 30].

**Table 1:** Species included in phylogenetic analyses, geographic origin of specimens, sample size and collecting depth for species of *Patagonotothen*

Species	Origin (Sample size)	COI GenBank	Collecting depth (meters)
*Eleginops maclovinus*	Beagle Channel (4)	N/A*	–
*Harpagifer bispinis*	Beagle Channel (3)	N/A	–
*Notothenia coriiceps*	South Shetland Islands (2)	N/A	–
*Trematomus eulepidotus*	South Shetland Islands (3)	MG770024-MG770025	–
*Lepidonotothen larseni*	South Shetland Islands (3)	MG770008-MG770010	–
*Lepidonotothen nudifrons*	South Shetland Islands (4)	MG770011-MG770014	–
*Lepidonotothen squamifrons*	Shag Rocks (5)	MG770015-MG770019	–
*Lepidonotothen kempi*	Shag Rocks (1)	N/A	–
	Bouvet Island (2)	MG770020-MG770021 MG770022-	–
	South Orkney Islands (2)	MG770023	–
*Patagonotothen sima*	Beagle Channel (6)	MG769992-MG769995	< 1
*Patagonotothen jordani*	Atlantic Patagonian Shelf (4)	MG770004-MG770007	36
*Patagonotothen elegans*	Atlantic Patagonian Shelf (3)	MG770000-MG770003	57
*Patagonotothen cornucola*	Beagle Channel (2)	MG769996-MG769997	< 1
	Atlantic coast of TDF** (2)	MG769998-MG769999	< 1
*Patagonotothen* cf. *cornucola*	Eastern limit of Beagle Channel (3)	MG770026-MG770028	30
*Patagonotothen canina*	Atlantic Patagonian Shelf (3)	MG769956-MG769959	77
*Patagonotothen tessellata*	Beagle Channel (7)	MG769948-MG769955	< 1
*Patagonotothen trigramma*	Beagle Channel (1)	N/A	6
*Patagonotothen guntheri*	Atlantic Patagonian Shelf (5)	MG769960-MG769962	100–128
	Shag Rocks (3)	MG769963-MG769964	309
*Patagonotothen brevicauda*	Atlantic Patagonian Shelf (5)	MG769965-MG769969	52–84
*Patagonotothen ramsayi*	Atlantic Patagonian Shelf (12)	MG769970-MG769983	60–100
	East of Burwood Bank (3)	MG769976-MG769978	309
*Patagonotothen wiltoni*	Beagle Channel (8)	MG769984-MG769991	6–10

*Non Available; **Tierra del Fuego

GenBank accession numbers for mitochondrial COI sequences are indicated

### ML phylogeny of Lepidonotothen and Patagonotothen

**Fig. 2:**
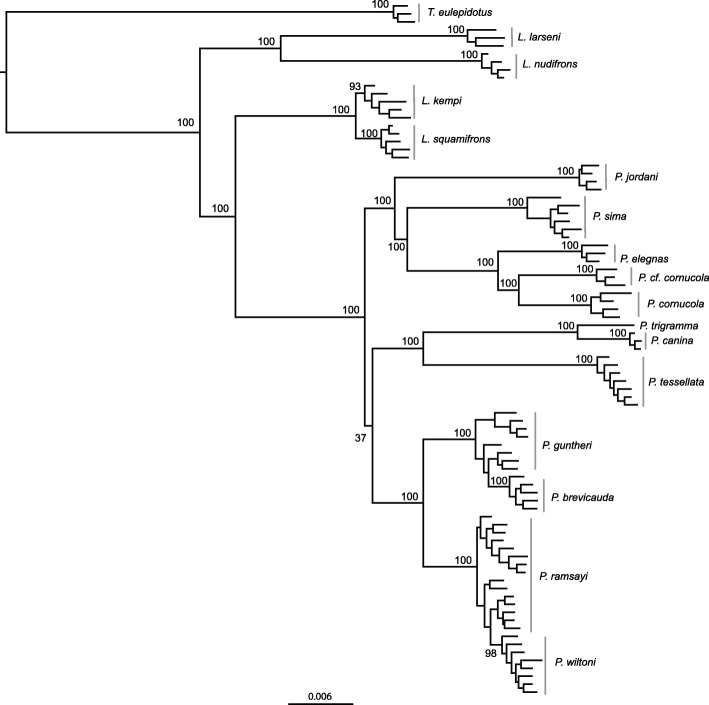
Maximum-Likelihood tree based on 18,485 SNPs from 2914 RAD loci. Distant outgroups (*E. maclovinus*, *H. harpagifer* and *N. coriiceps*) were excluded in order to maximize the recovered number of loci and SNPs. Node labels represent bootstrap support values as obtained with RAxML. Bootstrap support values within species were omitted for clarity (See full tree in Additional file 2: Figure S2)

To analyze the more recent speciation events, we performed a phylogenetic analysis with a separate data set excluding the distant outgroups *E. maclovinus*, *H. bispinis* and *N. coriiceps* to maximize the recovered number of loci and SNPs and thus to improve the resolution of the tree. In this case, Stacks recovered 2914 RAD loci across samples, harbouring a total of 18,485 SNPs (2.5% missing data allowed). We performed a ML phylogenetic analysis with all *Trematomus* (these were used as outgroup), *Lepidonotothen* and *Patagonotothen* samples (*n* = 87) (Fig. 2 and Additional file 3), and recovered strong bootstrap support (> 95) for most nodes in the resulting phylogeny. This analysis resulted in the following four key observations with implications for species delimitation:

(I). Evidence for a new *Patagonotothen* species: samples morphologically identified as *P. cornucola* (Richardson 1844) [31] following Brickle et al. [24] formed two divergent reciprocally monophyletic groups. These groups were also recovered using mtDNA (see below). We consider this to be good evidence for the presence of an unrecognized *Patagonothen* species, given that the level of divergence is comparable to the one observed between other well-established *Patagonotothen* species (Fig. 2). We therefore named the clade comprising the samples from the eastern limit of the Beagle Channel *Patagonotothen* cf. *cornucola*.
(II). The samples from *P. brevicauda* formed a monophyletic group nested within the paraphyletic *P. guntheri* clade.
(III). The samples of *P. wiltoni* formed a monophyletic group nested within the paraphyletic *P. ramsayi* clade.
(IV). Despite the close morphological similarity of *L. kempi* and *L. squamifrons* [32, 33], samples assigned morphologically to these species formed two reciprocally monophyletic groups.

In order to verify the paraphyly observed in *P. guntheri* and *P. ramsayi* with a model accounting for Incomplete Linage Sorting (ILS) we implemented a Polymorphism-Aware Phylogenetic Model. In the resulting topology both species also appear as paraphyletic (Additional file 4).

### Analysis of closely related species pairs using STRUCTURE

**Fig. 3:**
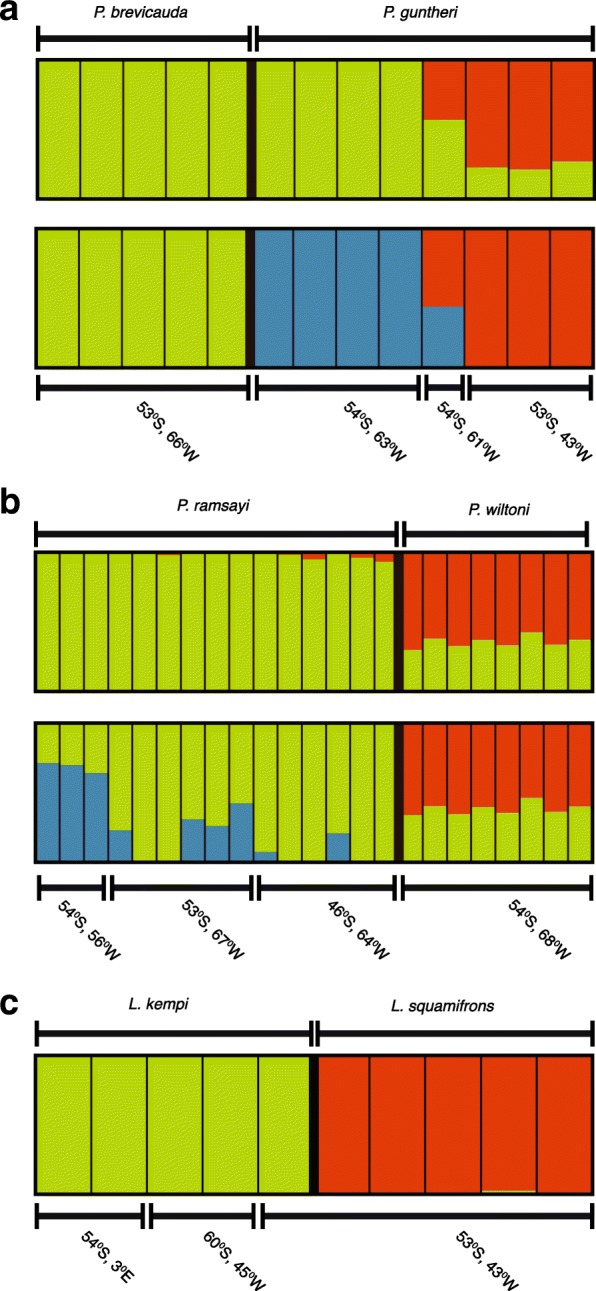
Analysis of differentiation between closely related species pairs using the software STRUCTURE based on genomic SNPs obtained through RADseq and assuming two (top) or three (bottom) inferred clusters. Below the plots the geographic origin of samples is indicates in latitude and longitude coordinates. **a** *P. breviauda* / *P. guntheri* species-pair differentiation inferred from a total of 2466 SNPs. **b** *P. ramsayi* / *P.wiltoni* species-pair differentiation inferred from a total of 3458 SNPs. **c** L. kempi / L. squamifrons species-pair differentiation inferred from a total of 999 SNPs

To further investigate differentiation between the closely related pairs of species *P. guntheri* – *P. brevicauda, P. ramsayi* – *P. wiltoni,* and *L. squamifrons* – *L. kempi*, we generated input files for STRUCTURE using the *population.map.pl* component of the Stacks pipeline, including a single randomly selected SNP from each RAD locus and tolerating no missing data. The results of the STRUCTURE analyses are shown in Fig. 3. For the species pairs *P. guntheri* – *P. brevicauda* and *P. ramsayi* – *P. wiltoni*, the most likely number of genetic clusters (K) was identified to be 2 or 3. We have considered both since biological meaningful information was observed. When assuming two clusters (K = 2) within the *P. brevicauda* – *P. guntheri* pair some samples of *P. guntheri* are identical to *P. brevicauda*; however, when assuming K = 3, one cluster correspond exclusively to the *P. brevicauda* samples and the other two to the *P. guntheri* samples, with one individual showing an admixed genotype. A substantial level of genetic admixture is also present in the *P. wiltoni* – *P. ramsayi* clade when assuming K = 2, although the two species appear clearly differentiated. Further genetic heterogeneity is revealed within *P. ramsayi* when assuming three genetic clusters (K = 3). For the *L. kempi* – *L. squamifrons* pair, the most likely value of the number of genetic clusters was 2. STRUCTURE was able to completely separate the species.

### Time-calibrated phylogeny inference with SNAPP

**Fig. 4:**
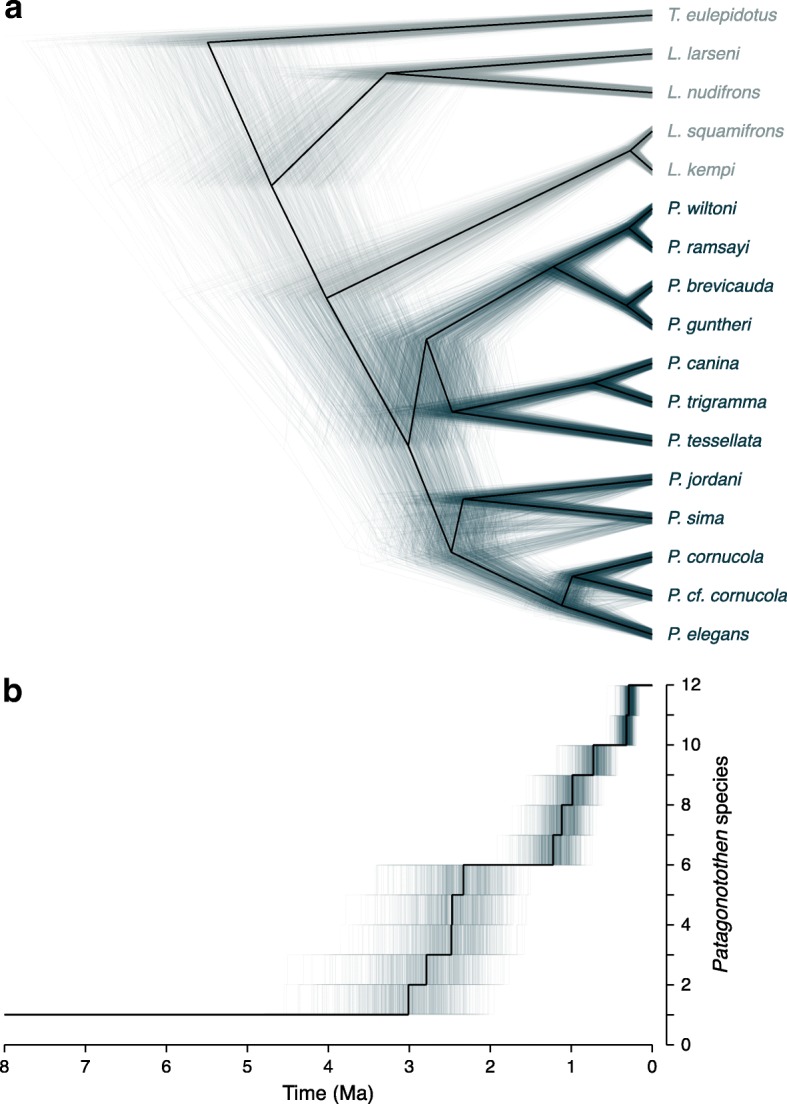
**a** Time-calibrated phylogeny generated with the software SNAPP based on 2778 SNPs. Three samples were included per species in the analysis except for *P. trigramma* from which only one specimen was available. **b** Lineage accumulation though time for *Patagonotothen* species

Three samples per species were selected for the SNAPP analysis except for *P. trigramma* from which only one specimen was available. For the species pairs *P. guntheri* – *P. brevicauda* and *P. ramsayi* – *P. wiltoni,* we considered the ML tree and the STRUCTURE results to select samples in order to capture most of the diversity within the species pairs. With these conditions Stacks generated an output file with 2778 SNPs. The SNAPP tree topology (Fig. 4a) was identical to the ML topology (Fig. 2) except for the relative positions of *P. sima* and *P. jordani*. Whereas *P. jordani* diverges earlier than *P. sima* in the ML tree, these two species appeared as sister taxa in the SNAPP phylogeny. The node supporting their sister-group relationship in the SNAPP phylogeny had a relatively low posterior support (0.703), probably related to the short internal branch leading to this speciation event. Figure 4b depicts the rate of speciation events and shows a remarkable period of more than one million years without reconstructed speciation events between approximately 2.3 Ma to 1.2 Ma ago.

### Mitochondrial locus

**Fig. 5:**
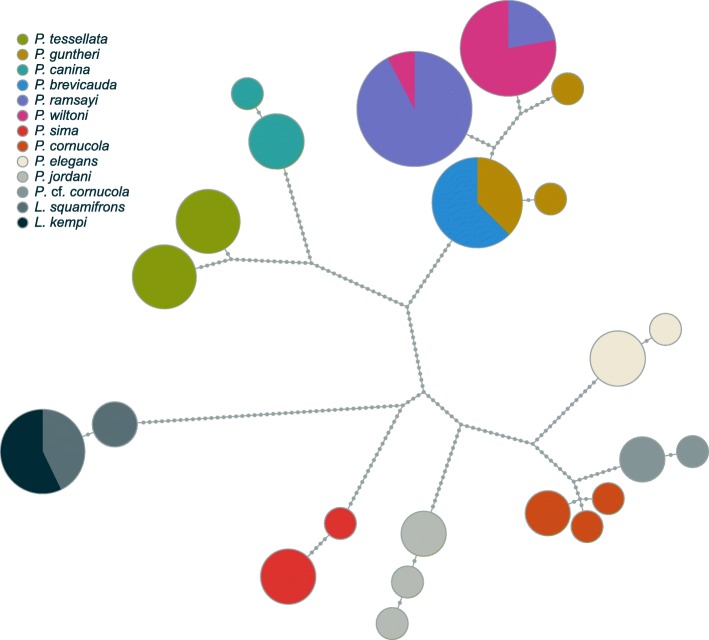
Mitochondrial haplotype genealogy generated with the software Fitchi based on a maximum-likelihood tree (Additional file 3: Figure S3). Haplotypes differing by a single nucleotide were collapsed for clarity

Maximum-Likelihood and the NJ reconstructions of the Cytochrome Oxidase I (COI) phylogeny resulted in a similar topology (Fig. 5 and Additional files 5 and 6); however, many nodes in the ML mitochondrial phylogeny had low bootstrap support (< 70). The mitochondrial locus failed to resolve the taxonomic identity of *P. brevicauda* and *P. guntheri* since the corresponding haplotypes did not form two reciprocally monophyletic clades. Despite a relatively high *F*_ST_ between *P. wiltoni* and *P. ramsayi,* we found three haplotypes shared between them (Fig. 5, Table 2). More specifically, two individuals of *P. ramsayi* had a COI haplotype found in most *P. wiltoni* samples, and the opposite was true for one *P. wiltoni* individual. In addition, the mitochondrial haplotype genealogy and phylogeny (Fig. 5 and Additional files 5 and 6) do not make clear that *P. wiltoni* and *P. ramsayi* are more closely related to one another than to either *P. guntheri* or *P. brevicauda* as is revealed by the nuclear phylogeny. On the other hand, *P. cornucola* and *P.* cf. *cornucola* still appeared as sister species and were well discriminated with the mitochondrial locus in accordance with the nuclear analysis. Regarding the *Lepidonotothen* species pair *L. kempi* – *L. squamifrons*, the mitochondrial locus was unable to resolve species identity in contrast to the RADseq data.

**Table 2:** Comparison of pairwise *F*_ST_ between genomic and mitochondrial markers in pairs of closely related species

Pairwise *F*_ST_		
Species pair	Nuclear markers (Loci)	Mitochondrial marker
*P. ramsayi / P. wiltoni*	0.015 (8849)	0.63
*P. guntheri / P. brevicauda*	0.027 (8540)	0
*P. cornucola / P.* cf. *cornucola*	0.17 (7307)	0.78
*L. squamifrons / L. kempi*	0.037 (9270)	0.085

The number of nuclear RAD loci used for the *F*_ST_ calculation is indicated in brackets

## Discussion

The main goal of this work was to generate a high-resolution phylogenetic framework for the genus *Patagonotothen* to better characterize the diversity of this group and to obtain insights into the processes that drove the radiation of this genus. The parameters used for the de novo assembly and filtering of loci performed well as demonstrated by the analysis including replicated samples (following the method suggested in reference [34]) and by the plot of loci shared by 80% of samples (Additional file 1), but also by the general agreement of the results with previous molecular phylogenies that show, for example, the paraphyly of the family Nototheniidae and the genus *Lepidonotothen* [5, 6, 27, 30]. At a finer taxonomic resolution, our phylogenetic results are generally in agreement with morphological differences, the geographic origin and/or the mitochondrial haplogroup of the samples.

### Taxonomic implications

#### Previously unrecognized linage

**Fig. 6:**
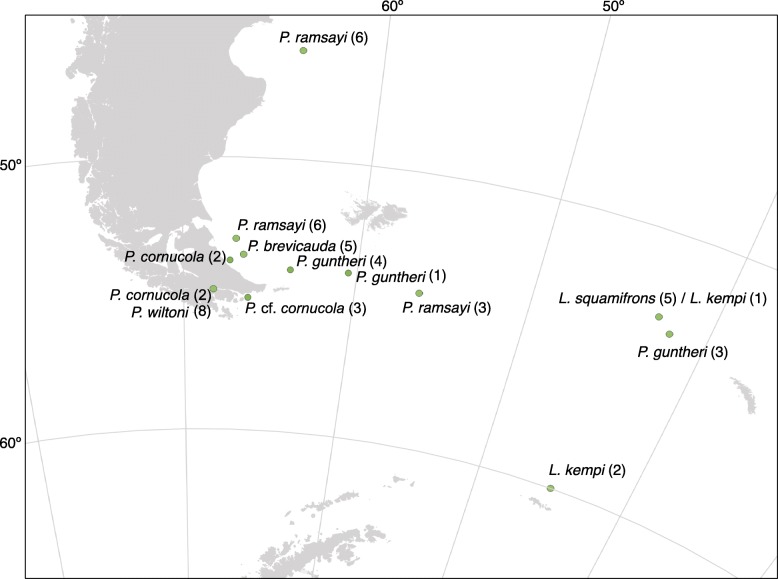
Map of Southern South America and northern tip of the Antarctic Peninsula showing the geographic origin of samples used for the STRUCTURE analysis of closely related species pairs and for *P. cornucola* and *P.* cf. *cornucola*. The number of samples per site is indicated in brackets. Only two samples of *P. kempi* collected near Bouvet Island (approximately 54^0^S, 3°E) are not shown in the map. The map was generated using the software QGIS v3.2.0

The individuals morphologically identified as *P. cornucola* (Richardson 1844 [31]) following Brickle et al. (2005) [24] formed two well-differentiated groups in our analyses, both based on genome-wide SNPs and a mitochondrial marker (Figs. 2 and 6). These groups have an estimated divergence time of ~ 1 Ma (Fig. 4). The level of genetic divergence between these two lineages (Table 2) is comparable to the divergence of any of them to the next-closest species *P. elegans*, or between other morphologically well-differentiated pairs of species like *P. ramsayi* – *P. guntheri* or *P. canina* – *P. trigramma* (Fig. 4a). In addition, geographic distance is unlikely to account for the separation of the two lineages because the samples of one clade were collected in the Beagle Channel (Bahía Golondrina, Ushuaia) and on the Atlantic coast of Tierra del Fuego (Auricosta), whereas the samples of the other clade were collected halfway between these two sampling points near the eastern limit of the Beagle Channel (Table 1 and Fig. 6). However, the individuals forming the former clade were caught at the intertidal zone, whereas the individuals of the second clade were caught at a depth of 30 m. This difference in collecting depth between these clades suggests spatial divergence at an ecological scale between the two lineages. Together, our results strongly support the recognition of the samples morphologically identified as *P. cornucola* as two genetically distinct, yet morphologically similar species.

Very little is known about the biology of *P. cornucola*. Most of the few studies carried out so far on *P. cornucola* have collected the individuals at the intertidal zone or in very shallow waters [19, 35–37]. In addition, the original description of the species by Richardson (1844) [31] states that specimens were collected “among the sea-weed that lines the shores of Cape Horn”, suggesting that the individuals were fished at very shallow waters. Thus, we used the *P. cornucola* denomination for the clade formed by the samples caught at the intertidal zone of Tierra del Fuego and *Patagonotothen* cf. *cornucola* for the species collected at the eastern limit of the Beagle Channel. Future studies should attempt to identify morphological differences between these two species to determine whether they are indeed morphologically indistinguishable and could therefore be considered cryptic, as has been previously suggested for Antarctic notothenioids species within the genus *Lepidonotothen* [38]. Until a morphological description of *Patagonotothen* cf. *cornucola* becomes available, the two species can be diagnosed by their distinct COI haplotypes (Table 1).

#### *The P. guntheri – P. brevicauda species pair*

The taxonomic identity of these two closely related species could not be resolved with mtDNA (*F*_ST_ = 0) and little differentiation was found across all genome-wide SNPs (mean *F*_ST_ = 0.027). In addition, the specimens of *P. guntheri* did not form a monophyletic group in the ML phylogeny (Fig. 2) and the genetic clustering conformed more to geography than morphology (Fig. 3a and Fig. 6). This is supported for K = 2 (STRUCTURE) in Fig. 3, where the Patagonian Shelf population of *P. guntheri* appeared to be closer to the *P. brevicauda* from the Patagonian Shelf than to the Shag Rock samples of *P. guntheri*. However, the single *P. guntheri* sample caught at Namuncurá - Burdwood Bank, which is geographically in between the other two sampling locations of *P. guntheri*, showed mixed membership (Fig. 3a and Fig. 6), suggesting a possible pattern of isolation by distance. Balushkin et al. (2000) [12] proposed that the Shag Rocks population of *P. guntheri* should be considered a different species (*P. shagensis*). Our analyses of mitochondrial and nuclear markers rather argue for *P. guntheri* from Shag Rock to be a different geographic population, or a subspecies, rather than a different species, similar to the case of *P. brevicauda*.

Morphologically, *P. brevicauda* and *P. guntheri* are very similar. The main diagnostic morphologies allowing their distinction are the depth of the caudal peduncle and the number of gill rakers (Norman, 1937). It has been suggested that *P. guntheri* usually occupies deeper waters than *P. brevicauda,* thus providing as similar case to the one found in *P. ramsayi* and *P. wiltoni* (Norman, 1937). Different evolutionary scenarios may account for the pattern found for *P. brevicauda* and *P. guntheri,* and only a broad population sampling could allow determining the genetic relationship between these two closely related species. Note that one of the *P. guntheri* samples (sample code: gun128 in the Additional files 5 and 6) has a mitochondrial haplotype that is closely related to the *P. wiltoni* mitochondrial clade; however, nuclear SNPs place this sample in a nested position within the *P. guntheri* clade, indicating that recent introgression may have occurred between *P. wiltoni* and *P. guntheri*.

#### *The P. wiltoni – P. ramsayi species pair*

We found a rather complex scenario in this species pair. While separated well at the mitochondrial marker (*F*_ST_ = 0.63), our genome-wide SNPs suggested a much weaker differentiation between *P. wiltoni* and *P. ramsayi* (mean *F*_ST_ = 0.015). In spite of the high *F*_ST_ value observed for mtDNA, both species share mitochondrial haplotypes (Fig. 5), suggesting past or ongoing hybridization. The STRUCTURE analysis also suggested a substantial level of shared genetic variation between the two species. In addition, the paraphyly of *P. ramsayi* as well as the heterogeneity observed with STRUCTURE (assuming K = 3) suggested that the level of differentiation between some *P. ramsayi* specimens is comparable to the differentiation found between *P. ramsayi* and *P. wiltoni*. Divergence into separate refugia during glaciation, possibly followed by admixture during postglacial periods, may be a potential explanation for these patterns. The simplest explanation for the much greater differentiation at the mitochondrial marker may be related to the four times lower effective population size in mitochondrial sequences compared to nuclear loci.

From a morphological standpoint, *P. wiltoni* and *P. ramsayi* are very similar. *P. wiltoni* can be distinguished from *P. ramsayi* by the smooth scales on the upper surface of the head, a larger mouth and smaller eyes, a narrower interorbital region, smaller average numbers of gill-rakers, a lower spinous dorsal fin, and generally darker color [25, 26]. Regarding habitat use, *P. wiltoni* is usually found in shallower waters than *P. ramsayi* [25, 26]. A wider geographic sampling would be needed to explore the extent of genetic differentiation between these two species as well as possible adaptive differences or reinforcement mechanisms.

Both species belong to the *longipes*-species group within the genus *Patagonotothen* as defined by Balushkin (1976) [see also 12]. Two other species belonging to this group as well are (*i*) *P. kreffti,* which appears to be most closely related to *P. ramsayi* and is mainly differentiated in the length of the interorbital space [26], and (*ii*) *P. longipes*, which is closely related to *P. wiltoni*, differing mildly in the length of the pelvic fin [25, 26]. It has even been suggested that *P. wiltoni* and *P. longipes* should be treated as synonyms for the same species (Norman, 1937). Unfortunately, we were not able to include *P. kreffti* and *P. longipes* in our analysis. However, given the low degree of genetic differentiation between *P. wiltoni* and *P. ramsayi*, we would expect even weaker differentiation between *P. wiltoni* and *P. longipes* or between *P. ramsayi* and *P. kreffti*. Future investigations should include these species to shed light on their taxonomic status.

#### *The L. kempi – L. squamifrons species pair*

The mitochondrial locus failed to resolve taxonomic identity between these species, contrasting our results from the genome-wide RADseq analysis. Our results based on mtDNA are in line with those of Miya et al. [39] who found low genetic differentiation between these two species based on mitochondrial markers. Furthermore, *L. kempi* specimens formed a clade nested within *L. squamifrons* in Miya et al. [39], which led the authors to propose that these may constitute geographic populations of the same species because the investigated *L. kempi* and *L. squamifrons* samples were obtained at sites separated by a large geographic distance. The same conclusion was reached by the study of Schneppenheim et al. [33] based on enzyme electrophoresis. In our analysis, two of the *L. kempi* specimens were collected near Bouvet Island, two near the South Orkneys, but one specimen was collected near Shag Rocks in exactly the same place as all *L. squamifrons* samples (Fig. 6). Therefore we can rule out the possibility that the differentiation found at genome-wide SNPs is simply due to isolation-by-distance. Our insights from nuclear SNPs are, despite their caveats due to small sample sizes, consistent with the hypothesis that *L. kempi* and *L. squamifrons* represent two separate species. The lack of differentiation found in mtDNA could be attributed to a low discriminating power of this single locus analysis. A wider geographic sampling, ideally including *L. kempi* and *L. squamifrons* individuals from the same geographic origin, and a genome-wide analysis will be needed to determine whether or not these two species should be lumped into one.

### Diversification timing and climatic history

Our time-calibrated phylogeny suggests that the *Patagonotothen* radiation proceeded in a shorter period of time than previously thought (~ 3 versus 5 My respectively [6]). Furthermore, the lineages-through-time plot (Fig. 4b) indicates that the rate of species accumulation may not have been constant but rather stepwise. An early burst of diversification roughly coincides with the onset of the intensification of Quaternary glacial cycles about 2.5 Ma [40]. A second round of speciation burst started around 1 Ma and coincides with the Greatest Patagonian Glaciation [41]. Finally, the three most closely related species pairs considered in our work (*P. wiltoni* – *P. ramsayi*, *P. guntheri* – *P. brevicauda* and *L. kempi* – *L. squamifrons*) show strikingly synchronous divergence times around 300 kya (Fig. 4). This dating falls within the Marine Isotope Stage 8, a period characterized by global glacial advance that would have been particularly extensive in Patagonia [42]. Thus, according to our calibration, the main burst of diversification could be related to major climatic events in the region during the Quaternary, which would have shaped and probably promoted the *Patagonotothen* radiation. One possible mechanism that could have driven this diversification is isolation and differentiation into separate refugia followed by expansion and secondary contact. On the other hand, glaciation may have generally exacerbated the extinction rate, resulting in vacant niches that could have provided ecological opportunities for speciation. For example, glaciations are expected to affect inshore and coastal fauna to a greater extent while having a smaller effect on species that inhabit deeper waters. *P. wiltoni*, *P. brevicauda* and *P. cornucola* may therefore have originated from their deeper-living counterparts (*P. ramsayi*, *P. guntheri* and *P.* cf. *cornucola,* respectively) following the ecological opportunities provided as a consequence of the retraction of glaciers in near shore or coastal areas. Whether the divergence was mainly promoted by geographic isolation during glaciation, by ecological opportunity following the retreat of glaciers, or by intrinsic biological factors remains an open question.

Recent studies have shown that per capita speciation rates are currently higher in the temperate and cold zone than the tropics whereas the rates were higher in the tropics in the past, but there is little insight into the underlying mechanisms of speciation [43, 44]. The further study of the high latitude and recent radiation of *Patagonotothen* may contribute important data to understand how the latitudinal gradient in species diversity has developed and how it may evolve, a key frontier for future research in marine organisms [44].

## Conclusion

This study represents the most extensive genome-wide phylogenetic analysis of the genus *Patagonotothen*. First, we demonstrate that RADseq is effective in gathering thousands of genome-wide markers to resolve phylogenetic relationships in the Antarctic notothenioid clade, including some of its oldest divergence events. We further show that the genetic boundaries between some species in the genus are diffuse, especially between *P. brevicauda* and *P. guntheri,* and between *P. ramsayi* and *P. wiltoni*. Some levels of recent introgression and/or insufficient time for fixation of alternative nuclear and mitochondrial alleles between the species may explain these results. We also provide genetic evidence for the presence of a new, potentially cryptic, *Patagonotothen* species. A deeper population-level sampling will be needed to refine these patterns and underlying processes in this ongoing radiation in which species are likely to have diverged very recently and may now be experiencing secondary contact.

Our time-calibrated phylogeny shows a period spanning more than one million years (representing more than a third of the age of the *Patagonotothen* clade) without species accumulation, suggesting that the rate of species accumulation was not constant but rather stepwise, probably shaped by major climatic events during the Quaternary. Future studies should address the underlying speciation mechanisms and the relative importance of putative allopatric divergence during glaciations and ecological opportunity afterwards.

Our phylogenetic analysis should serve as a reference for future investigations of the diversification and systematics within the genus. This first step in understanding the *Patagonotothen* radiation, a group of fish with Antarctic ancestry that have radiated in a sub-Antarctic environment, will also be important for insights into the possible fate of the spectacular diversity of Antarctic notothenioids in a warming Southern Ocean.

## Methods

### Sampling and molecular methods

In total, we collected 67 specimens belonging to twelve *Patagonotothen* species (Table 1) in southern Patagonia by means of three different methods: (*I*) by bottom-trawling onboard the Oceanographic Vessel Puerto Deseado and the RV Polarstern, (*II*) by using trammel nets in the archipelago Islas Bridges at the Beagle Channel and (*III*) by capturing fish by hand in the intertidal zone during low tide in the Beagle Channel and in the Atlantic coast of Tierra del Fuego. When fishes were captured alive they were euthanized using buffered tricaine methanesulfonate (MS-222) or Eugenol (clove oil). Individuals were determined to the species level following Brickle et al. [24], originally adapted from Norman [25]. A piece of white muscle tissue was preserved from each individual in 90% EtOH and stored at − 20 °C until subsequent analysis. In our analysis, we further included the following species as phylogenetic outgroups: *Eleginops maclovinus*, *Harpagifer bispinis*, *Notothenia coriiceps*, *Lepidonotothen larseni*, *L. nudifrons*, *L. squamifrons* and *L. kempi,* leading to a total of 96 individuals (Table 1).

Genomic DNA was extracted from about 20 mg of muscle tissue using the DNeasy Blood and Tissue Kit (Qiagen). We then prepared three libraries of individually barcoded RAD [45], following the protocol described in Roesti et al. [46, 47], adopted from Hohenlohe et al. [48]. In short, each sample was individually subjected to restriction digestion using the *Sbf1* enzyme, followed by the fusion of a specific 5-mer barcode to the restricted DNA of each individual. Subsequently no more than 40 individuals were pooled into a sequencing library. Two individual samples were included twice in different libraries for a posterior evaluation of the de novo assembly performance as suggested by Mastretta el al. [34]. Each library was single-end sequenced to 100 bp reads in three separate lanes on an Illumina HiSeq2500 at the Quantitative Genomics Facility, D-BSSE, in Basel (Switzerland).

In addition, approximately 700 bp of the mitochondrial cytochrome oxidase I (COI) gene were amplified by polymerase chain reaction (PCR) and subsequently Sanger-sequenced. For the PCR, we used the forward primer COX1L5928D 5’-TCRACYAAYCAYAAAGAYATYGGCAC-3′ and the reverse primer COX1H6664D 5’-TAKACYTCWGGGTGDCCRAARAAYCA-3′ in a volume of 30 μl, containing 15 μl of total DNA (4 μg/ml), 1 unit of Taq DNA polymerase (Promega), 1x Taq polymerase buffer, dNTPs (0.2 mM of each), forward and reverse primers (0.3 mM of each) and MgCl_2_ (2.5 mM). The following cycling conditions were applied on a 2720 Thermal Cycler (Applied Biosystems): an initial denaturation of 3 min at 94 °C, 35 cycles of 30 s of denaturation at 94 °C, 30 s of annealing at 50 °C, and 1 min of extension at 72 °C, followed by a final extension of 5 min at 72 °C. PCR products were then sequenced in both directions at Macrogen, South Korea. Chromatograms were scored and analyzed using BioEdit v7.2.5 [49].

### Assembly of reads into RAD loci and variant calling

We conducted a de novo assembly of RAD loci with the software Stacks, version 1.41 [28]. Raw sequences were demultiplexed according to individual barcodes and filtered for low quality using the *process_radtags* module. From each sequence, we trimmed the 5-bp barcode and the remainder of the Sbf1 enzyme recognition site, yielding a final sequence read length of 89 bp. We then used the wrapper program *denovo_map.pl* to execute the components of the Stacks pipelines *ustacks*, *cstacks* and *sstacks,* followed by the *rxstacks* error correction module and, finally, a second iteration of *cstacks* and *sstacks*. The pipeline first aligns reads at the individual level and makes stacks with perfectly matching sequences when a minimum coverage is reached. This is controlled by the parameter m (minimum stack depth) that was set to 6. Afterwards, based on the number of nucleotide differences between stacks, the software merges putative alleles together into a locus. This is controlled with parameter M (distance allowed between stacks) that was set to M = 3. Subsequently, a catalogue of all loci and alleles across individuals was generated and the software merged stacks up to a threshold distance that is controlled by the parameter n, which was set to n = 3. Finally, we exported SNPs (including IUPAC encoded ambiguities for heterozygous sites), using the program *populations* from the Stacks pipeline. We excluded loci absent in more than 5% of the total samples and loci that did not reach a minimum coverage depth of 12 per individual.

During the preparation of the manuscript we became aware of the protocol suggested in reference [29] for parameters optimization in Stacks. Consequently, we followed this protocol to assess the performance of the parameters we have used (especially M = 3 and n = 3 as described above). We selected 10 samples, 2 from each of five species of the genus *Patagonotothen* that are representative of the variation within the genus (*P. jordani*, *P. tessellata*, *P. cornucola*, *P. ramsayi* and *P. guntheri*). We then iterated the *denovo_map.pl* module of Stacks varying M from 1 to 6 and fixing M = n, and plotted the number of loci shared by at least 80% of the samples.

### Phylogenetic and population genomics analysis

Maximum-likelihood trees were generated using RAxML (v8.2.1) [50] assuming a GTR + GAMMA model with the Lewis ascertainment bias correction on the CIPRES Science Gateway platform [51]. To account for possible incomplete lineage sorting in the relatively young radiation of *Patagonotothen* species, we also estimated the species tree with the software SNAPP [52]. We combined the Bayesian species-tree inference in SNAPP with divergence-time estimation based on the model of Stange et al. [53]. In brief, this model employs a strict molecular clock, links the population sizes of all extant and ancestral lineages, and assumes a Jukes-Cantor [54] model of sequence evolution. To time-calibrate the molecular clock, we applied log-normal age constraints according to the divergence times inferred by Colombo et al. [8], on the initial divergence of Trematomini (mean in real space: 6.0 Ma; standard deviation: 0.165 Ma) and on the most recent common ancestor of *Lepidonotothen* and *Patagonotothen* (mean in real space: 4.58 Ma; standard deviation: 0.25 Ma). To reduce computational cost, we performed the SNAPP analysis with a data set that contained no missing data and included only 3 samples per species and a single randomly selected SNP per RAD locus (see section 3.5). Input files for SNAPP were prepared with the script snapp_prep.rb (https://github.com/mmatschiner/snapp_prep). We performed four replicate SNAPP analyses with the same input file. Each replicate had a chain length of 1 million Markov-chain Monte Carlo (MCMC) iterations, of which the first 100,000 iterations were discarded as burnin. The samples generated by the four chains were merged into a single posterior distribution of parameter estimates and trees. A maximum-clade-credibility (MCC) tree was generated from the posterior tree sample using TreeAnnotator of the BEAST2 v2.4.3 software package [55].

We applied a population genomic approach to assess the level of differentiation between closely related species pairs within *Patagonotothen* using the Bayesian method of Pritchard et al. [56] implemented in the software STRUCTURE 2.3.4. Runs were performed with the number of genetic clusters (*K*) fixed between 1 and 4 using an admixture model with correlated allele frequencies. Six independent runs were conducted for each *K* value. Preliminary runs showed that convergence was achieved after 50,000 iterations. Thus, this was used as burn-in and the estimations were based on 100,000 additional iterations. We inferred the *K* values that best captured the structure of the data by plotting the ‘Ln Probability of Data’ against *K* as suggested by the software developers; however, more than one *K* value were considered for subsequent analysis when biological meaningful information was observed. Results of STRUCTURE analyses were plotted with the script bar_plotter.rb (http://evolution.unibas.ch/salzburger/software/bar_plotter.rb).

The genealogy of the mitochondrial locus was inferred using RAxML (v8.2.1) [50] assuming a GTR + GAMMA model on the CIPRES Science Gateway platform [51] and using a NJ algorithm as implemented in MEGA 6.0.6 [57]. A mitochondrial haplotype-genealogy graph was generated with Fitchi [58], using the phylogenetic tree obtained with RAxML as input.

## Additional files

Additional file 1: Plot for assembly parameters selection. (PDF 882 kb)

Additional file 2: ML tree with all samples. (PDF 1485 kb)

Additional file 3: ML tree with *Lepidonotothen* and *Patagonotothen* samples. (PDF 1162 kb)

Additional file 4: Polymorphism-Aware Phylogenetic Model. (PDF 903 kb)

Additional file 5: ML tree for the partial sequences of COI. (PDF 1274 kb)

Additional file 6: NJ tree for the partial sequences of COI. (PDF 1260 kb)

## Abbreviations

AFGPs: Antifreeze glycoproteins
COI: Cytochrome Oxidase I
ILS: Incomplete Linage Sorting
ML: Maximum-Likelihood
NJ: Neighbour-Joining
RADseq: Restriction site-associated DNA sequencing
SNPs: Single nucleotide polymorphisms
