# Supplementary material for: Phylogenomics of an extra-Antarctic notothenioid radiation reveals a previously unrecognized lineage and diffuse species boundaries
Source: BMC Evol Biol. 2019 Jan 10;19:13. doi: 10.1186/s12862-019-1345-z (PMC6327445; doi:10.1186/s12862-019-1345-z)
Supplement: Supplementary file 1 — Plot for assembly parameters selection. (PDF 882 kb) [file 12862_2019_1345_MOESM1_ESM.pdf]

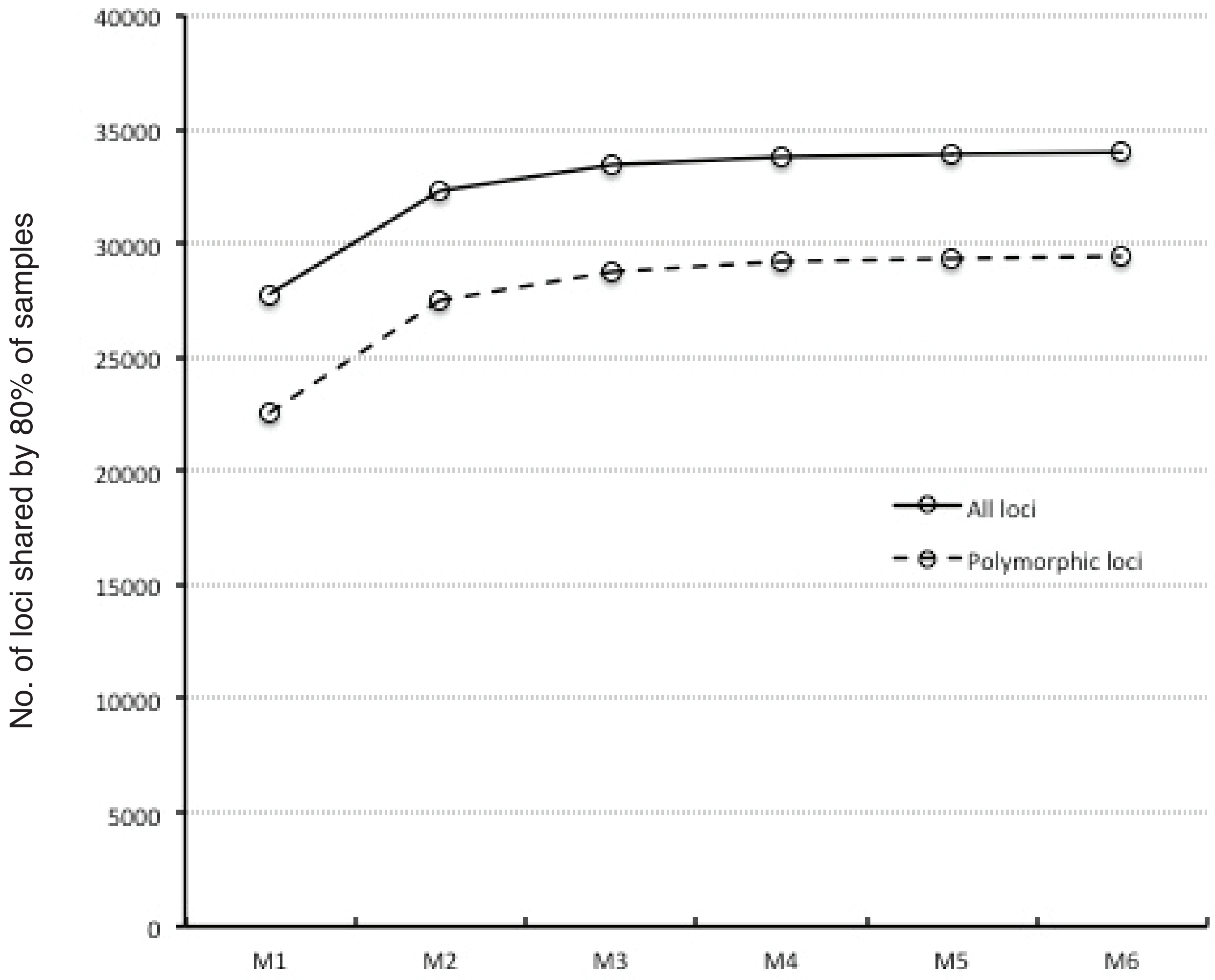


**Additional file 1**. Selection of assembly parameters in the *de novo* analysis. Increasing the M and n assembly parameters, and fixing M = n, affects the number of loci (solid line) and polymorphic loci (dashed line) shared by 80% of samples or more. Note that for M and n values ≥ 3 the number of loci is mostly unaffected. This analysis was done in a subset of ten representative samples of the variation within the genus *Patagonotohen*.
