# Supplementary material for: Phylogenomics of an extra-Antarctic notothenioid radiation reveals a previously unrecognized lineage and diffuse species boundaries
Source: BMC Evol Biol. 2019 Jan 10;19:13. doi: 10.1186/s12862-019-1345-z (PMC6327445; doi:10.1186/s12862-019-1345-z)
Supplement: Supplementary file 3 — ML tree with Lepidonotothen and Patagonotothen samples. (PDF 1162 kb) [file 12862_2019_1345_MOESM3_ESM.pdf]

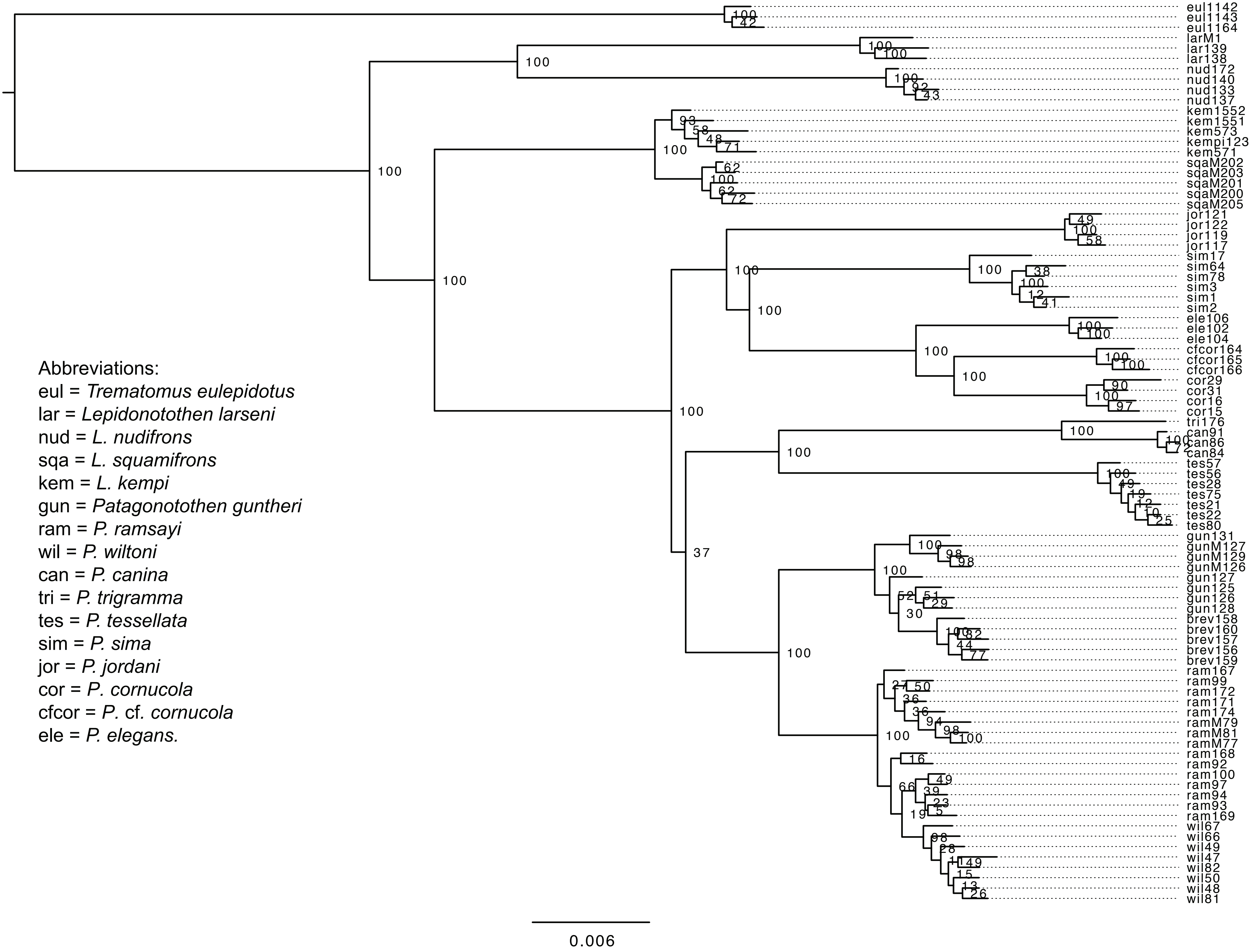

Additional file 3. Maximum-likelihood tree based on 18,485 SNPs from 2,914 RAD loci. Distant outgroups (*E. maclovinus, H. harpagifer* and *N. coriiceps*) were excluded in order to maximize the recovered number of loci and SNPs. Node labels represent bootstrap support values as obtained with RAxML.
