# Supplementary material for: Phylogenomics of an extra-Antarctic notothenioid radiation reveals a previously unrecognized lineage and diffuse species boundaries
Source: BMC Evol Biol. 2019 Jan 10;19:13. doi: 10.1186/s12862-019-1345-z (PMC6327445; doi:10.1186/s12862-019-1345-z)
Supplement: Supplementary file 4 — Polymorphism-Aware Phylogenetic Model. (PDF 903 kb) [file 12862_2019_1345_MOESM4_ESM.pdf]

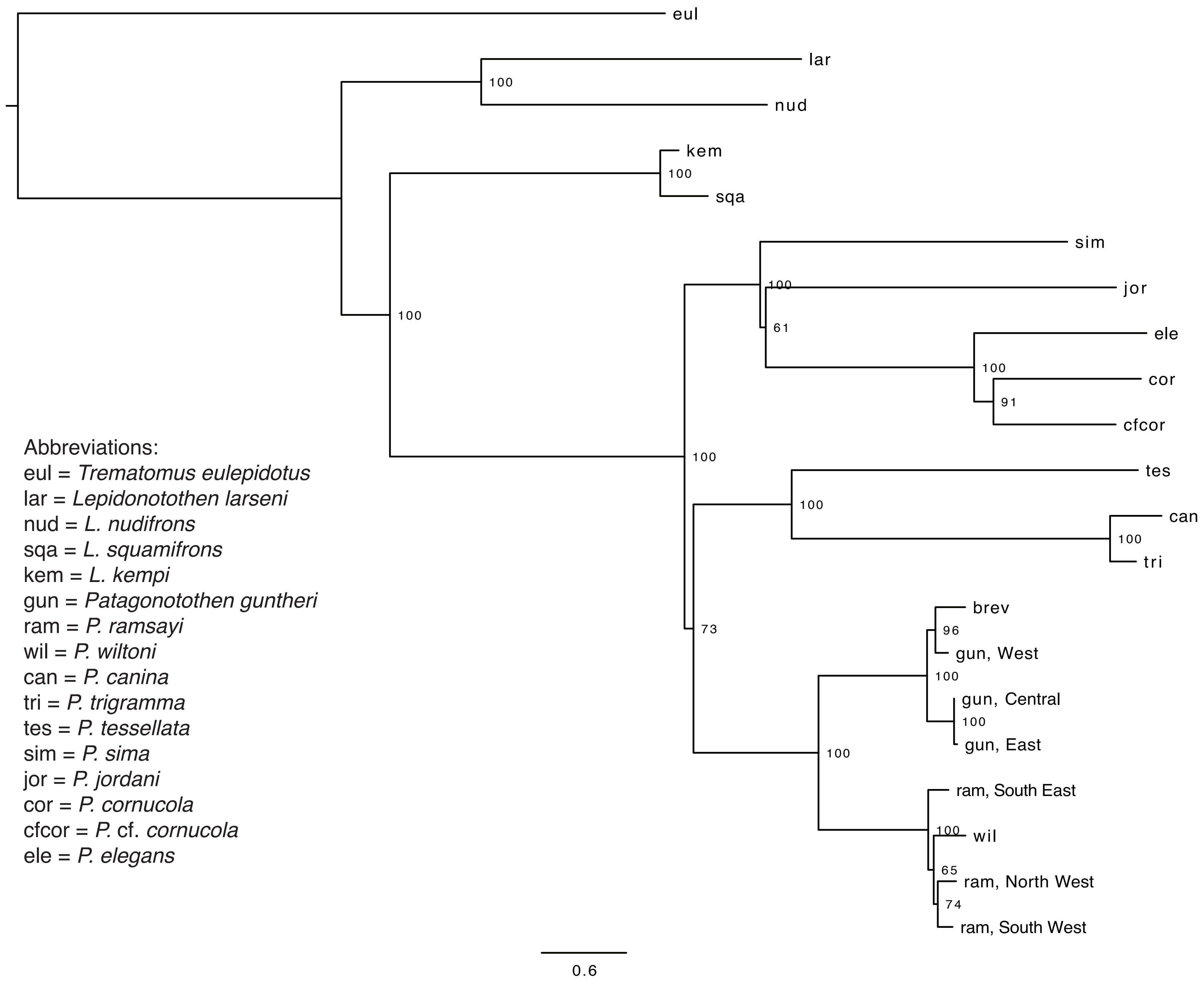

Additional file 4. Phylogenetic inference resulting from the application of the Polymorphism-Aware Phylogenetic Model as implemented in the maximum likelihood software IQ-TREE. Individual samples were grouped by species and by geographic origin in the case of *P. ramsayi* and *P. guntheri*. Node labels represent bootstrap support values.
