# Supplementary material for: Phylogenomics of an extra-Antarctic notothenioid radiation reveals a previously unrecognized lineage and diffuse species boundaries
Source: BMC Evol Biol. 2019 Jan 10;19:13. doi: 10.1186/s12862-019-1345-z (PMC6327445; doi:10.1186/s12862-019-1345-z)
Supplement: Supplementary file 5 — ML tree for the partial sequences of COI. (PDF 1274 kb) [file 12862_2019_1345_MOESM5_ESM.pdf]

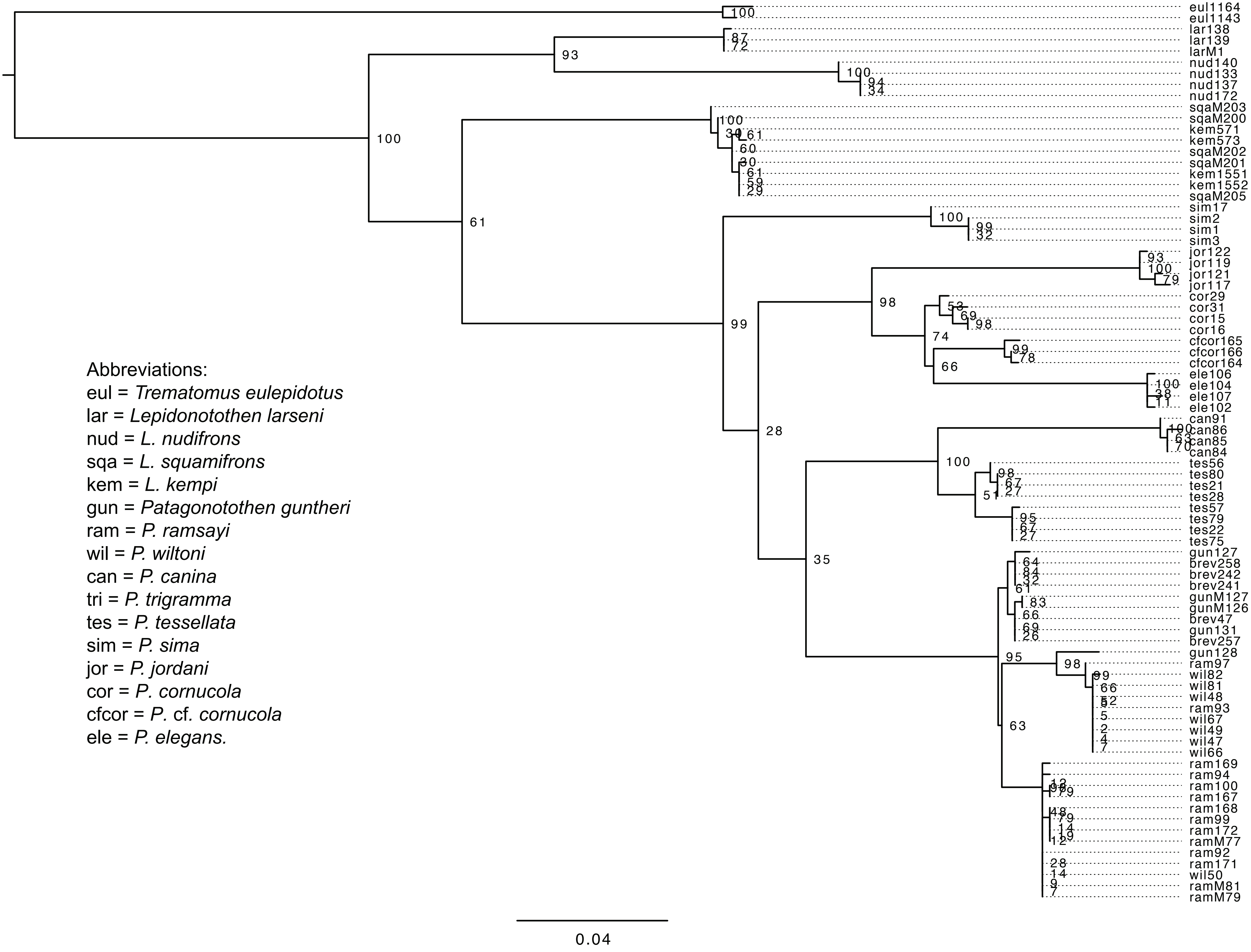

Additional file 5. Maximum Likelihood tree based on COI sequences of 620 bp. Node labels represent bootstrap support values as obtained with RAxML.
