# Supplementary material for: Phylogenomics of an extra-Antarctic notothenioid radiation reveals a previously unrecognized lineage and diffuse species boundaries
Source: BMC Evol Biol. 2019 Jan 10;19:13. doi: 10.1186/s12862-019-1345-z (PMC6327445; doi:10.1186/s12862-019-1345-z)
Supplement: Supplementary file 6 — NJ tree for the partial sequences of COI. (PDF 1260 kb) [file 12862_2019_1345_MOESM6_ESM.pdf]

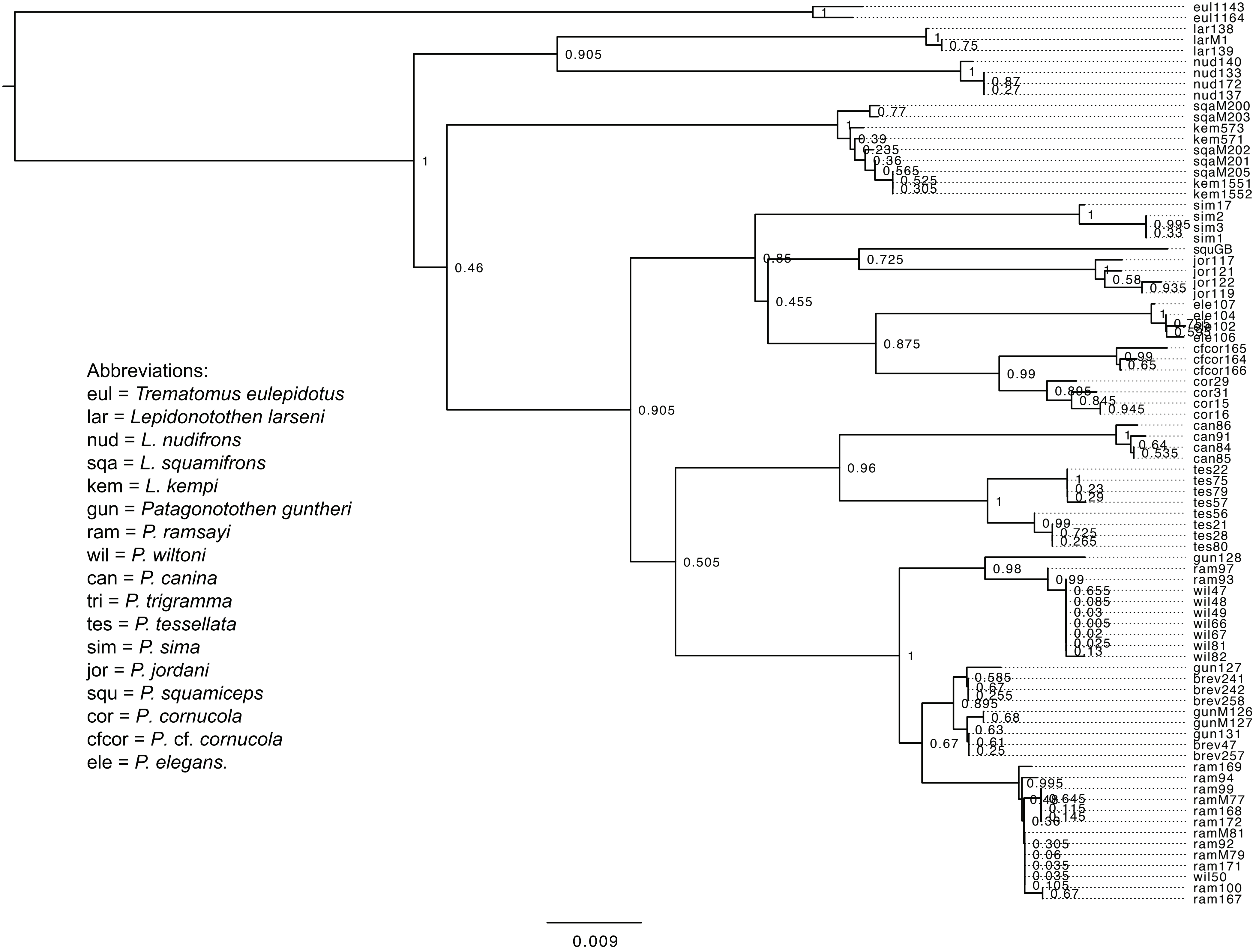

Additional file 6. Neighbour Joining tree based on COI sequences of 620 bp. Node labels reprensent bootstrap support values as obtained with MEGA 6.06.
