# Supplementary material for: Phylogenomics of an extra-Antarctic notothenioid radiation reveals a previously unrecognized lineage and diffuse species boundaries
Source: BMC Evol Biol. 2019 Jan 10;19:13. doi: 10.1186/s12862-019-1345-z (PMC6327445; doi:10.1186/s12862-019-1345-z)
Supplement: Supplementary file 2 — ML tree with all samples. (PDF 1485 kb) [file 12862_2019_1345_MOESM2_ESM.pdf]

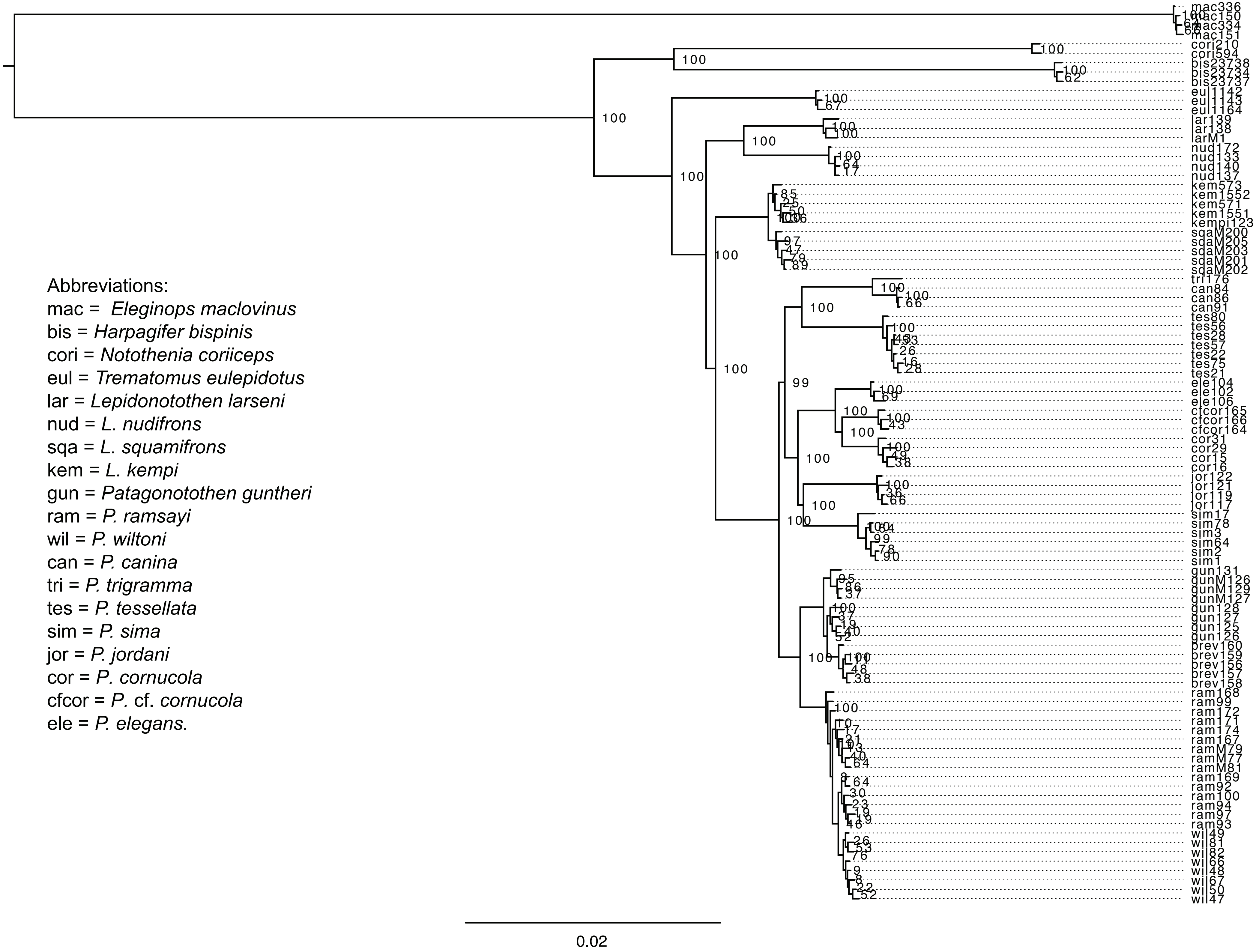

Additional file 2. Maximum-likelihood tree based on 11,804 SNPs from 1,682 RAD loci including distant outgroups to assess early diversification in notothenioids. Node labels represent bootstrap support values as obtained with RAxML.
